# Barriers and facilitators to qualitative data sharing in the United States: A survey of qualitative researchers

**DOI:** 10.1371/journal.pone.0261719

**Published:** 2021-12-31

**Authors:** Jessica Mozersky, Tristan McIntosh, Heidi A. Walsh, Meredith V. Parsons, Melody Goodman, James M. DuBois

**Affiliations:** 1 Bioethics Research Center, Division of General Medical Sciences, Washington University School of Medicine, St. Louis, MO, United States of America; 2 School of Global Public Health, New York University, New York, NY, United States of America; University of Toronto, CANADA

## Abstract

Qualitative health data are rarely shared in the United States (U.S.). This is unfortunate because gathering qualitative data is labor and time-intensive, and data sharing enables secondary research, training, and transparency. A new U.S. federal policy mandates data sharing by 2023, and is agnostic to data type. We surveyed U.S. qualitative researchers (N = 425) on the barriers and facilitators of sharing qualitative health or sensitive research data. Most researchers (96%) have never shared qualitative data in a repository. Primary concerns were lack of participant permission to share data, data sensitivity, and breaching trust. Researcher willingness to share would increase if participants agreed and if sharing increased the societal impact of their research. Key resources to increase willingness to share were funding, guidance, and de-identification assistance. Public health and biomedical researchers were most willing to share. Qualitative researchers need to prepare for this new reality as sharing qualitative data requires unique considerations.

## Introduction

Qualitative health data—such as data from interviews or focus groups—are rarely shared in the United States (U.S.) [1 p.161, 2]. This is unfortunate because qualitative data are labor and time-intensive to gather, and data sharing would enable secondary research, enhance training, and increase transparency. In contrast, qualitative data sharing is more common in places such as the UK, Finland, Germany, and Australia [3, 4]. The UK Data Service is now a well-established archive providing infrastructure and services to facilitate qualitative data sharing with a collection of nearly 1000 data sets [3].

The National Institutes of Health (NIH), the largest federal funding body of health research in the U.S., recently updated its policy for data management and sharing to increase data sharing obligations and enforcement. The policy will take effect in 2023. NIH guidance states that, ‘data should be made as widely and freely available as possible while safeguarding the privacy of participants, and protecting confidential and proprietary data’ [5]. The policy is agnostic to data types, defining data broadly as ‘recorded factual material commonly accepted in the scientific community as necessary to validate and replicate research findings’ including unpublished data [5]. This policy is noteworthy because funded researchers frequently share quantitative data to comply with NIH policy, but the revised policy will require data sharing of all types of data, potentially leaving qualitative researchers unprepared for the coming reality.

Our prior work has identified a series of benefits and concerns regarding qualitative data sharing (QDS). It could increase the transparency of research and enable verification of findings, which can foster public trust [1, 2, 6–8]. Sharing data enables secondary users to explore new research questions, or collate findings across multiple studies, maximizing the value of data that are often costly and resource intensive to collect. QDS may reduce participant burden by allowing researchers to use existing data rather than collect new data. QDS also provides an opportunity for students to learn how to conduct data analysis, examining research questions using real data when they have no funding to gather their own [1, 3, 4, 9–14].

However, in healthcare settings, qualitative researchers often investigate sensitive or stigmatized issues with vulnerable participants [10, 15]. Given that qualitative data are often sensitive, qualitative researchers have expressed concerns about informed consent, protecting confidentiality, maintaining trust and relationships, and ensuring appropriate secondary uses of data, if data were shared [1–3, 10, 16, 17]. Some argue that the information disclosed by participants is only made possible because of the trusting relationship between researcher and participant [1, 18, 19]. Researchers fear that sharing qualitative data could undermine this trust and that participants may be prevented from providing full and honest disclosure if they know data are going to be shared. In addition, participants may consent to have their data interpreted for one purpose, not for secondary purposes by a different researcher.

Qualitative data are non-numeric, which poses an additional de-identification challenge because identifiers may be located anywhere within long passages of narrative text [20]. Currently, researchers must manually search for and locate identifiers within qualitative data during data cleaning and analyses, but the process is labor intensive and we lack tools to support researchers in this process. Adequate de-identification of qualitative data requires balancing the protection of individual identities while ensuring adequate contextual detail remains to enable secondary use. In concurrent work, we are developing automated support software to assist researchers in the de-identification process [21].

Data repositories can also help address researcher concerns about QDS. Repositories store, preserve, and manage data in a manner that enables data sharing, discovery, and citation [3, 22, 23]. Repositories are staffed with experts who can help with data curation, provide guidance on preparing data for deposit, and work with researchers to determine an appropriate level of restriction for their data, including restricted access and delayed access options for secondary users [24]. Secondary users of sensitive data typically sign a data use agreement, which stipulates that they will not attempt to identify participants and they must obtain Institutional Review Board (IRB) approval prior to receiving data [25]. The data use agreement is brokered by the repository.

When adopting a new and controversial practice, it is important to engage stakeholders to understand the facilitators and barriers to uptake, and to promote stakeholder buy-in for the new practice [26, 27]. In this article, we report findings from a survey of qualitative researchers regarding their experience and attitudes toward sharing health related or sensitive qualitative data in a repository where other researchers could access data. Qualitative data are gathered in diverse fields, including public health, social work, anthropology, medicine, occupational and physical therapy, nursing, bioethics, psychology, and clinical research [28]. Hence, we recruited broadly across health-related fields to ensure broad representation from qualitative researchers. We aimed to identify researchers’ top concerns, and factors that might increase their willingness to share.

The survey is part of a larger project to identify and overcome practical and ethical barriers to QDS in the U.S. (R01HG009351-01A1). In the next phase of our research, we will conduct a formative evaluation trial with 30 qualitative researchers that involves using our newly developed de-identification support software and guidelines prior to depositing their qualitative research data set with our partner repository, the Inter-university Consortium for Political and Social Research (ICPSR) at the University of Michigan. All survey participants were invited to take part in the formative evaluation QDS pilot at the end of the survey. The survey aimed to answer four research questions:

**RQ #1:** How supportive are qualitative researchers of sharing qualitative data with a repository?
**RQ #2:** What exploratory variables are associated with overall attitudes toward qualitative data sharing?
**RQ #3:** What are the most common concerns that qualitative researchers have about sharing their qualitative data with a repository?
**RQ#4:** What are the most common considerations and resources that would make qualitative researchers more willing to share their qualitative data with a repository?

## Materials and methods

### Survey development

Prior to survey development, formative in-depth interviews were conducted with 30 qualitative researchers to inform survey content [2]. During these interviews, researchers were asked about the practical and ethical barriers and facilitators of sharing qualitative data with a repository. Transcripts from these interviews were coded for perceived barriers and concerns, as well as perceived benefits and facilitators of sharing qualitative data. Interviewees expressed a wide range of concerns and identified several benefits of sharing qualitative data with a repository. These data and prior literature on QDS guided the development of the survey items [1, 14, 19, 29–31].

### Recruitment

This study used a non-probability, criterion-based sampling approach. A non-probability approach was necessary because there is no way to identify the number nor identity of all qualitative researchers. Criterion-based sampling was used to target appropriate informants: Individuals who conduct qualitative research in the U.S. We restricted our focus to the U.S. because regulations, oversight policies, and data sharing practices might all affect attitudes, and these vary across nations.

Qualitative researchers were contacted via email through a range of recruitment mediums. We identified publicly available contact information of investigators through NIH RePORTER by using the search terms ‘qualitative,’ ‘interview,’ and ‘focus group.’ We also searched academic institution websites and recruited through the listservs of professional organizations for qualitative researchers in general (e.g., Society for Medical Anthropology). To ensure adequate representation from minority groups, we recruited through professional organizations for researchers from minority communities (e.g., Robert Wood Johnson Foundation New Connections Network, Brothers of the Academy, Sisters of the Academy, Latina Researchers Network) [32]. We also used a word of mouth approach, in which we contacted colleagues through our professional networks to request they send the recruitment email to potential qualitative researchers.

An email was distributed to qualitative researchers with a request to take the survey via an anonymous survey link. We asked professional organizations and academic institutions to send the link through their listservs and post the study information and anonymous survey link on their social media accounts (e.g., Twitter or Facebook). The survey was administered using Qualtrics survey software, which is password and firewall protected. The research was approved as exempt by the Institutional Review Board at Washington University (IRB#201811123). Participants who completed the survey were entered into a raffle to win one of ten cash prizes worth $100 for participating.

### Data analysis

Data were analyzed using Stata statistical software (version 16.0); statistical significance is assessed as p<0.05. Bivariate analyses were conducted using Pearson’s chi-squared test to explore which demographic variables are associated with overall attitudes toward QDS within this sample.

## Results

### Sample

Of the 676 respondents who initiated the web-based survey, 251 were excluded from analyses for not meeting study criteria (i.e., participants had not led and conducted qualitative research with human subjects, participants did not work at an institution in the U.S.) or did not complete more than 50% of the survey, which was forced-choice to avoid the problem of missing data. The final sample comprised of 425 qualitative researchers in the U.S. from a variety of academic disciplines including: public health, bioethics, and clinical fields (i.e., medicine, nursing, occupational and physical therapy) (n = 152, 38%); anthropology and sociology (n = 133, 33%); and other disciplines (n = 118, 29%). Table 1 presents the demographic information describing the sample. The majority of participants were female (n = 324, 76%), White (n = 242, 57%), and between 30–49 years old (n = 260, 61%).

**Table 1:** Demographic information of survey respondents (N = 425).

Demographic Questions	Percent	Count
Do you ever collect both quantitative data and qualitative data in the same research project?		
Yes	87%	370
No	13%	55
Do you ever gather health information in your qualitative research, such as information about a participant’s diagnoses, symptoms, or treatments?		
Yes	71%	301
No	29%	124
Do you ever gather other forms of sensitive information, such as information about illegal behaviors and sexual behaviors?		
Yes	48%	206
No	52%	219
Which of the following methods do you use to gather qualitative research data? (check all that apply)		
Interviews	98%	415
Focus groups	76%	321
Observations	61%	261
Archival research	39%	165
Town hall meetings or other deliberative methods	22%	94
Other	14%	59
Who has funded your research in the last 5 years? (check all that apply)		
Your institution	74%	314
Government agencies	65%	276
Private foundations	48%	202
Other	8%	35
Corporations	5%	22
Have you ever shared your qualitative research data with people outside of your research team?		
No	80%	340
Yes	20%	85
Which of the following best describes how you shared your qualitative research data? (check all that apply)*		
Shared data with an individual outside of your research team	76%	65
Deposited data with a data repository or archive	18%	15
Other	18%	15
If you have shared your qualitative data, what was the reason for doing so? (check all that apply)*		
To encourage new uses for my data	59%	50
To promote new research collaborations	58%	49
To show a commitment to openness	33%	28
To increase the impact and visibility of my research findings	31%	26
Other	25%	21
A requirement of a funding agency	16%	14
A requirement of a journal	6%	5
A requirement of the institution	7%	6
To shift the burden of maintaining and archiving my data to the repository	2%	2

*N* = 425.

*Question only applies to participants who have shared qualitative data previously (*N* = 85).

Most qualitative researchers indicated they had been conducting qualitative research for more than 10 years (n = 235, 55%) and collect health information (n = 301, 71%) and other sensitive information (n = 206, 48%) in their qualitative research. Participants reported conducting research with various populations, including pregnant women (n = 85, 20%), children (n = 130, 31%), prisoners (n = 13, 3%), sexual minorities (n = 101, 24%), individuals with cognitive impairments (n = 47, 11%), those older than 65 (n = 144, 34%), patients (n = 203, 48%), racial and ethnic minorities (n = 333, 78%), economically disadvantaged people (n = 286, 67%), and individuals with sensitive diagnoses (n = 135, 32%). A variety of qualitative methods were used by respondents, the most common being interviews (n = 415, 98%) and focus groups (n = 321, 76%). Their work has been funded by government agencies (n = 276, 65%), private foundations (n = 202, 48%), corporations (n = 22, 5%), and investigator’s institutions (n = 314, 74%).

### Experience and overall attitudes toward qualitative data sharing

The vast majority of researchers (n = 410, 96%) have never shared qualitative data in a repository. Qualitative researchers were asked to rate on a seven-point Likert scale the extent to which they oppose or support sharing qualitative research data (1 = strongly oppose, 7 = strongly support). Attitudes about sharing qualitative data were mixed. Specifically, 41% (n = 174) of participants oppose sharing qualitative data (including strongly oppose and slightly oppose), and 49% (n = 208) of participants support QDS (including strongly support and slightly support), indicating a bimodal distribution of attitudes toward sharing qualitative data with a repository.

Participants’ field of study was significantly related to attitudes toward QDS, such that those who conducted research in public health, clinical fields (e.g., nursing and medicine), and bioethics were more likely to support sharing qualitative data (*M* = 4.52, *SE* = .16) than those who conducted research in disciplines of anthropology or sociology (*M* = 3.58, *SE* = .17). Participant race was significantly associated with attitudes toward QDS. Hispanic (*M* = 4.40, *SE* = .32) and Asian (*M* = 4.48, *SE* = .39) qualitative researchers tended to be more supportive of sharing qualitative data compared to their White (*M* = 4.16, *SE* = .13) and Black (*M* = 3.94, *SE* = .22) qualitative researcher counterparts.

Age was associated with opposition to sharing qualitative data with a repository. Generally, younger qualitative researchers (e.g., age 20–29, *M* = 4.00, *SE* = .38; age 30–39, *M* = 4.08, *SE* = .16) tended to be less supportive of sharing qualitative data than qualitative researchers who were older (e.g., age 50–59, *M* = 4.21, *SE* = .22; 60+, *M* = 4.39, *SE* = .26). Finally, participant sex was associated with attitudes toward sharing qualitative data, such that males (*M* = 4.81, *SE* = .21) were more supportive of sharing qualitative data than females (*M* = 4.04, *SE* = .11).

### Interest in qualitative data sharing pilot study

All survey participants were asked if they were interested in participating in our pilot study that involves using newly created de-identification support software on a qualitative data set prior to deposit in a data repository. Here, we treat interest in participating in the pilot as a measure of researcher willingness to share qualitative data. Out of 425 qualitative researchers, 134 (32%) expressed interest in participating in the pilot. Bivariate analyses indicate that collecting sensitive qualitative data (*p* = 0.046), the sex of the researcher (*p* = 0.006), and prior sharing experience (*p* = 0.019) are significantly associated with interest in participating in the pilot. Of those researchers who gather sensitive information (n = 206), 36% (n = 74) expressed interest in the pilot compared to 27% (n = 60) of those who do not gather sensitive information (n = 219). Men (n = 33, 45%) were more likely to express interest in participating in the pilot study compared to women (n = 98, 30%). Participants who reported sharing qualitative research data with people outside of their research team in the past (n = 85) were more likely to be interested in participating in the pilot study (n = 36, 42%) compared to those who have not shared data previously (n = 98, 29%).

### Concerns regarding qualitative data sharing

Qualitative researchers were asked how concerned they were about various factors related to sharing their qualitative data through a repository, on a scale of 1 (not at all concerned) to 5 (extremely concerned). Table 2 presents the frequencies of these concerns. Researchers’ greatest concerns (rated item a 3 or above) included that they lack participant permission (n = 370, 87%), data sensitivity (360, 85%), concerns about breaching participant trust (n = 349, 82%), IRB or institutional policies (n = 336, 79%), and inability to adequately de-identify data (n = 334, 79%).

**Table 2:** Concerns regarding qualitative data sharing.

How concerned are you about the following factors related to the idea of sharing qualitative data through a repository?	Frequency of Participants Who Indicated Concern (%)
Lack of permission from research participants to share data.	370 (87%)
The sensitivity of research data.	360 (85%)
Breach of trust with participants.	349 (82%)
IRB or institutional policies.	336 (79%)
Concern that data cannot be adequately anonymized.	334 (79%)
Losing control over who has access to my qualitative data.	326 (77%)
The time and effort to prepare data for deposit.	325 (76%)
The potential for misinterpretation of my data by other researchers.	316 (74%)
Financial cost to prepare qualitative data for deposit.	283 (67%)
Issues with legal permissions	252 (59%)
Potential for repository technology failure.	233 (55%)
My lack of knowledge about repositories and data sharing in general.	223 (52%)
Others do not deserve to use data I collected.	94 (22%)
I do not like the idea of others judging my work.	74 (17%)

Items were rated on a scale of 1 (not at all concerned), 2 (slightly concerned), 3 (moderately concerned), 4 (very concerned), or 5 (extremely concerned). Participants were considered to be concerned if they rated 3–5.

### Facilitators to qualitative data sharing

Participants were asked how likely certain considerations would increase their willingness to share qualitative data through a repository, on a scale of 1 (not at all likely) to 5 (very likely). Table 3 presents the frequencies of these considerations (rated item a 4 or above). Researchers indicated that they would be most likely to share their qualitative data if doing so increased the societal impact of their research (n = 353, 83%), if participants agreed to have their data shared (n = 339, 80%), and if sharing led to increased future collaborations (n = 322, 76%).

**Table 3:** Considerations that would increase willingness to share.

How likely would each of the following considerations increase your willingness to share qualitative data through a repository?	Frequency of Participants Willing to Share (%)
If sharing increased the societal impact of research.	353 (83%)
If I knew my participants would agree to data sharing.	339 (80%)
If sharing led to increased collaborations.	322 (76%)
If sharing decreased the burden on participant communities.	308 (72%)
If secondary data users needed to cite their data sources in all publications.	294 (69%)
If data could be reused to explore new research questions.	283 (67%)
If sharing made data from publicly-funded research more widely available.	286 (67%)
If repositories provided a secure infrastructure for data storage.	279 (66%)
If those who share data are invited to be co-authors on papers that use data.	275 (65%)
If funding agencies required data to be shared.	266 (63%)
If sharing helped avoid duplication of work.	260 (61%)
If sharing data created the opportunity for students to learn how to analyze data.	257 (60%)
If sharing allowed for verification of data interpretation.	230 (54%)
If sharing positively influenced career promotion decisions.	226 (53%)
If repositories provided a central catalog of available data sets.	214 (50%)
If sharing led to increased citations.	205 (48%)
If journals required data to be shared.	201 (47%)

The degree to which each consideration would increase willingness to share qualitative data were rated on a scale of 1 (not at all likely), 2 (somewhat unlikely), 3 (neutral), 4 (somewhat likely), or 5 (very likely). Participants were considered willing to share if they rated 4 or 5.

### Resources to facilitate qualitative data sharing

Qualitative Researchers were asked to rate, on a scale of 1 (not at all) to 5 (a great deal), how much certain resources would increase their willingness to share their qualitative data through a repository. Table 4 presents the frequencies of these resources (rated item a 4 or above). Participants indicated that they would be more willing to share their data if repository costs were covered by funding agencies (n = 294, 69%), if they received clear guidance on ethics and compliance-related issues (n = 259, 61%), if repositories assisted with data anonymization (n = 243, 57%), and if repositories provided consultations on sharing qualitative data (n = 207, 49%).

**Table 4:** Resources to facilitate data sharing.

How much would each of the following resources increase your willingness to share qualitative data?	Frequency of Participants Willing to Share (%)
If funding agencies would cover the cost of sharing qualitative data with a repository.	294 (69%)
If you were given clear guidance on ethics and compliance-related issues.	259 (61%)
If a data repository assisted with data anonymization.	243 (57%)
If a data repository provided consultations regarding sharing qualitative data.	207 (49%)

The degree to which each resource would increase willingness to share qualitative data were rated on a scale of 1 (not at all), 2 (a little), 3 (a moderate amount), 4 (a lot) or 5 (a great deal). Participants were considered willing to share if they rated a 4 or 5.

## Discussion

Findings from the current study indicate that QDS in the U.S. remains rare, with only 4% of qualitative researchers having ever shared qualitative data in a repository. While nearly half of researchers expressed support for QDS, most researchers are not actually sharing qualitative data currently. These findings, although focused on qualitative researchers in the U.S., have implications for qualitative researchers more broadly given the international shift towards data sharing and open science, including qualitative data, which has historically not been shared.

### Limitations

We used a criterion-based sampling approach which limits the generalizability of our findings. This non-probability approach was necessary because there is no way to identify all qualitative researchers, so our approach was to target appropriate informants. When individuals completed the entire survey, we had no missing data from them because we used forced choice; however, some individuals chose not to complete the survey after establishing eligibility. We do not know how those who completed the survey differ from non-responders. In addition, we restricted data collection to US qualitative researchers so our findings may not generalize to other national contexts with different legal and regulatory frameworks. Finally, we conducted analyses on the association of demographics (e.g., age, sex, and field of study) with attitudes toward data sharing and willingness to participate in our data sharing pilot project. These associations are “within sample” associations and should be interpreted as such.

### Resources and guidance needed

#### Clear and transparent consent forms

Researchers’ top concerns related to obtaining informed consent, ensuring participants agreed, and not breaching trust. Notably, in concurrent work, we conducted qualitative interviews with 30 qualitative research participants and found the majority supported QDS and some assumed data sharing was already happening [8]. Participants were broadly supportive of QDS so long as confidentiality was maintained and data were shared with other researchers. Going forward, qualitative researchers must ensure clear and transparent informed consent that communicates data sharing plans at the outset of a study as this could significantly facilitate QDS, and be acceptable to participants. Historically, qualitative researchers often promise in informed consent documents to destroy data when the research ends or that no one outside the research team will ever access data. These statements prohibit data sharing from the outset. While such statements may be appropriate in some cases where data are too sensitive to share or cannot be de-identified adequately, in many cases clear and transparent consent forms that obtain permission for data sharing will enable QDS going forward. We believe it is appropriate for consent forms to specifically disclose that secondary analyses may explore new research questions [33]. Importantly, such broad statements would pertain to secondary analyses conducted by third parties on shared data as well as analyses on new research questions conducted by the original investigators, which is the most common form of “secondary analyses” currently [33].

In addition, consent forms will need to specifically disclose that secondary analyses may be conducted once data is shared, and that such analyses may explore new or different research questions than originally planned [33]. A recent review of qualitative secondary analyses found that the majority were conducted by the original investigators involved in the parent study, primarily to explore new questions on a subset of existing data, and there was a lack of clarity when reporting on whether these analyses were an extension of the primary analyses or a secondary analyses [33]. Informed consent documents need to include a clearer differentiation of primary and secondary analyses, including that secondary analyses could explore topics entirely unrelated to the primary study [33].

#### Repositories

Qualitative researchers expressed concerns regarding losing control of who accesses data (77%), financial costs of preparing data (67%), concerns about potential repository technological failures (55%), and lack of knowledge of repositories and QDS in general (52%). At the same time, 66% of researchers indicated they would be more willing to share if repositories provided a secure infrastructure for data storage. We encourage researchers to explore available repositories, whether institutional or national, as appropriate repositories can provide the necessary tools and guidance to facilitate QDS such as archiving data securely (and in perpetuity) and restricting secondary users’ access to data. Restricted access, rather than public access, is likely appropriate for most types of sensitive qualitative health data. In the U.S., funders such as the NIH allow data sharing costs to be included in budgets, and researchers should confirm with their funders as QDS may be an allowable cost [5].

#### Assistance with de-identification

The majority of qualitative researchers (79%) expressed concerns that qualitative data cannot be adequately de-identified, and 59% reported that resources to assist with de-identification would enhance their willingness for QDS. Currently researchers must manually sift through data to look for and remove potential identifiers, which is labor intensive. In addition, there are no standards specific to qualitative data to determine when it is adequately de-identified. In concurrent work, we are developing automated software to assist qualitative researchers de-identifying qualitative data [21]. Such automated tools will facilitate de-identification prior to data sharing, although researcher input is still required to verify that data are adequately de-identified. It is also essential that data retain adequate contextual details to enable secondary users to interpret the data. Repositories can provide guidance on the necessary accompanying documentation and contextual data to enable secondary use.

#### Factors associated with willingness to share: An area for future research

Our data indicate that public health, clinical health, and bioethics researchers are more open to QDS than researchers from other fields such as anthropology and sociology. This may be partially due to the common use in anthropology and sociology of ethnographic methods such as participant observation that require detailed, and often deeply personal, field notes. These data may be especially difficult to de-identify and present greater challenges for sharing than a one-time qualitative interview conducted in a public health or medical research setting. Alternately, this may reflect the cultures in which public health, medical, and nursing researchers work and their funding sources [9]. In contrast to researchers in disciplines like anthropology who may return to field notes throughout their careers for analyses, researchers in medicine, nursing, and public health are more likely to conduct contract funded research with a clear end date [9] and funders may also require data sharing. These qualitative researchers often have data that may not have been ‘fully mined’ by the time research funding ends, creating opportunities for further analyses [9]. Biomedical and public health researchers may be prime candidates for championing and helping to normalize QDS.

Qualitative researchers’ age, sex, and race were associated with attitudes toward QDS in our sample, with men, those who are older in age, and Asians and Hispanics being more supportive of QDS compared to their counterparts. Given that our survey was not designed to determine why these factors are associated with attitudes towards sharing, future research is needed to better understand whether, how, and why individual factors may influence willingness to share. Future research should also examine what resources could help overcome barriers to sharing qualitative data with a repository outside of the U.S., as barriers will likely differ by national context.

### QDS is feasible and can improve healthcare

Qualitative data are often sensitive, provide rich information, and seek to explore complex inquiries not adequately addressed using quantitative methods. Qualitative insights have changed healthcare and practice, suggesting there is much unrealized potential if more qualitative data were shared. For instance, a systematic review of 77 original qualitative studies on a form of chronic pain led to a new understanding of pain as an ‘adversarial struggle’, illustrating that a central component of therapy is that patients must feel recognized and heard by physicians [13]. At the same time, the existence of nearly 80 studies on a similar topic suggests that qualitative research may at times be wasteful or duplicative; an avoidable occurrence if qualitative data were shared more broadly [11].

QDS has the potential to improve transparency, promote secondary data analysis, and facilitate research training, but researcher attitudes and behaviors need to change. In fact, researchers cited increasing the societal impact of their work and future collaborations as key factors that would increase their willingness to share. Realizing such goals will require actually changing behavior and long-held attitudes about QDS. The UK illustrates this potential for change. There has been a slow but steady rise in qualitative data sharing as a result of the open data movement, funding policies, changing attitudes, and the availability of practical procedures and ‘mature infrastructure’ through the UK Data Archive [3]. An analysis of 267 data sets in the UK Data Archive (not necessarily health related) indicates there were 7,155 unique downloads of these data sets. Data were primarily used for learning (64%), research (15%), and teaching (13%) and demonstrate the ‘scale and significance of the reuse of data for teaching and learning’[3].

Our current project aims to provide the necessary support and resources to facilitate QDS in the U.S., including developing a software to support the de-identification of qualitative data, and a QDS Toolkit containing guidance and materials. We are engaging diverse stakeholders to identify concerns and needs, develop and evaluate the Toolkit, and will disseminate the Toolkit to strategic groups while evaluating its adoption. At the end of the project, the Toolkit—including the software—will be made available to support data sharing in an ethical manner in the U.S.

The NIH is moving ‘toward a future in which data sharing is a community norm’[5], including sharing de-identified qualitative health data, and other funding agencies may soon follow suit. It is imperative that qualitative researchers increase their knowledge of how to share their qualitative research data responsibly. Widespread, responsible sharing of qualitative data can have a lasting positive impact on health knowledge and interventions [9, 11–13, 34, 35]. Systematic guidelines and support for responsible and ethical QDS are needed to realize the potential benefits while protecting confidentiality and maintaining trust among research participants and the research community. Some data, if released and not shared responsibly, could present real harm to participants. However, responsible sharing of qualitative health data is possible and would maximize the value and use of data for health, social science, and policymaking.

## Supporting information

S1 Data (XLSX)
